# Molecular Characterization, Expression Pattern, and Ligand-Binding Property of Three Odorant Binding Protein Genes from *Dendrolimus tabulaeformis*

**DOI:** 10.1007/s10886-014-0412-6

**Published:** 2014-04-12

**Authors:** Sufang Zhang, Zhen Zhang, Hongbin Wang, Xiangbo Kong

**Affiliations:** Key Laboratory of Forest Protection, Research Institute of Forest Ecology, Environment and Protection, Chinese Academy of Forestry, State Forestry Administration, No.1 Dongxiaofu, Haidian, Beijing, China

**Keywords:** Pheromone binding proteins, General odorant binding proteins, Real-time PCR, Fluorescence competitive binding assay, Forest insect, Economic pest, Lepidoptera

## Abstract

Odorant binding proteins (OBPs) play important roles in insect olfactory processes. The Chinese pine caterpillar moth, *Dendrolimus tabulaeformis* (Lepidoptera, Lasiocampidae) is a serious economic pest in China, and the pheromones of this species have been identified to monitor their presence. However, the molecular mechanisms by which *D. tabulaeformis* perceive pheromones and host volatiles remain unknown. In this study, we identified and characterized three new OBPs, including one pheromone binding protein (PBP1) and two general odor binding proteins (GOBPs), from antennal cDNA of *D. tabulaeformis*. The deduced amino acid sequences of *DtabPBP1*, *DtabGOBP1*, and *DtabGOBP2* revealed mature proteins of 140, 147, and 140 amino acids, respectively. Each has six cysteine residues in conserved positions relative to other known OBPs. Amino-acid alignments indicated that the two GOBPs are more conserved (DtabGOBP1 is 52.9–67.4 % identical to orthologs from other Lepidoptera, and DtabGOBP2 is 55.2–81.8 % identical) than the PBP (32.5–46.0 %). Real-time PCR indicated tissue- and sex-specific expression patterns of the three genes. *DtabPBP1* was mainly expressed in the antennae of males, whereas female antennae had only 1.09 % the expression in male antennae. Both *DtabGOBP1* and *DtabGOBP2* were more highly expressed in antennae than in other tissues, while *DtabGOBP1* was more abundant in male antennae and *DtabGOBP2* in female antennae. In addition, the binding specificities of the three proteins were investigated, and all three OBPs exhibited high binding affinities for the pheromone component (5*Z*,7*E*)-5,7-dodecadien-1-yl propionate (*Z*5,*E*7-12:OPr). This suggests a role in binding pheromone for GOBPs, as well as PBP1, in *D. tabulaeformis*.

**Electronic supplementary material:**

The online version of this article (doi:10.1007/s10886-014-0412-6) contains supplementary material, which is available to authorized users.

## Introduction

The Chinese pine caterpillar moth, *Dendrolimus tabulaeformis* (Lepidoptera, Lasiocampidae) is a serious economic pest in northern China (Yan 1992). In an outbreak, *D. tabulaeformis* larvae feed intensively on the needles of conifers, and their population density can reach 400 per tree (Li 2008). The resulting damage to the tree may reduce the seed yield, influence the growth of the tree, and even cause tree death (Liu et al. 2008). To date, aerial treatments with chemical and bacterial insecticides are the main methods of controlling *D. tabulaeformis*, and people even manually collect the cocoons during outbreak years. But these methods are either environmentally unfriendly or inefficient.

Chemical signals are the primary basis of interactions between insects and their surroundings, and olfaction is a major communication system in insect (de Bruyne and Baker 2008; Touhara and Vosshall 2009). Environmental volatiles, such as host plant volatiles, and pheromones from potential mates are two important kinds of chemical attractants for insects (Bruce et al. 2005; Karlson and Butenandt 1959). Recently, the sex pheromones of *D. tabulaeformis* were identified; they are (5*Z*,7*E*)-5,7-dodecadien-1-yl acetate (*Z*5,*E*7-12:OAc), (5*Z*,7*E*)-5,7-dodecadien-1-ol (*Z*5,*E*7-12:OH), and (5*Z*,7*E*)-5,7-dodecadien-1-yl propionate (*Z*5,*E*7-12:OPr), with an optimal ratio of 100:100:4.5 (Kong et al. 2012). The pheromones have been used to monitor the population dynamics of *D. tabulaeformis*. Terpenes are the most abundant volatiles from *Pinus tabuliformis*, the favorite host of *D. tabulaeformis* (Liu 2007), and the volatile mixtures from *P. tabuliformis* can elicit much greater electroantennogram (EAG) responses than those from non-host plants (Liu 2007). However, the molecular mechanisms by which *D. tabulaeformis* perceive pheromones and host volatiles remains unknown.

Sensilla of insect antenna are the primary airborne chemical detectors because they house the neuronal receptors for odorants. A detailed description of the morphology and ultrastructure of *D. tabulaeformis* sensilla has been given in our previous work (Zhang et al. 2013). Odorant binding proteins (OBPs) are soluble proteins in the sensillar lymph that are thought to participate in the first step of odor detection by specifically binding odor molecules and transporting them to receptors (Benton et al. 2007; Grosse-Wilde et al. 2006; Pelosi et al. 2006; Xu et al. 2005). The first OBP, also the first pheromone-binding protein (PBP), that was identified from *Antheraea polyphemus* (Lepidoptera: Saturniidae) (Vogt and Riddiford 1981). Since then, OBPs have been identified from various insect species (e.g., Garczynski et al. 2012; Gu et al. 2011; Guo et al. 2012; Krieger et al. 2004; Picimbon and Gadenne 2002; Vogt 2002; Xiu and Dong 2007; Xiu et al. 2008; Zhang et al. 2009; Zheng et al. 2013; Zhu et al. 2013). Among Lepidoptera, there are two important classes of OBPs, PBPs and general odorant binding proteins (GOBPs), with the GOBPs further classified into GOBP1 and GOBP2 subgroups (Gong et al. 2009b; Pelosi et al. 2006). The role of PBPs in binding pheromone components has been demonstrated in many species (Du and Prestwich 1995; Feixas et al. 1995; Maïbèche-Coisne et al. 1997). GOBPs have been proposed to bind a wide range of general odors such as plant volatiles, but with lower specificity than the PBPs for pheromone (Vogt et al. 1991a). However, recent reports indicate that GOBPs can also bind pheromone components in some moth species. For example, GOBP2 of *Orthaga achatina* (Butler) showed high binding affinities for their sex pheromones (Liu et al. 2012b). GOBP2 protein of *Chilo suppressalis* (Walker) had significant affinity to *Z*-11-hexadecenal (*Z*11–16:Ald), the main component of *Ch. suppressalis* pheromone and to two general plant volatile aldehydes (Gong et al. 2009b). GOBP2 of *Bombyx mori* can bind both bombykol and bombykal, with the binding affinities for these two odorants differing (Zhou et al. 2009). GOBP2 of the navel orangeworm, *Amyelois transitella* (Walker), binds the major component of the sex pheromone, (*Z-*11)(*Z-*13)-hexadecenal with high affinity (Liu et al. 2010). These findings indicate a complex function of PBP and GOBPs.

In addition to their different ligands, PBPs and GOBPs also exhibit different expression patterns: GOBPs are expressed largely in both male and female antenna (Krieger et al. 1996; Vogt et al. 1991a, 2002; Zhang et al. 2010), whereas PBPs show more complex expression patterns between the sexes. Initial research has indicated that PBPs are expressed only in males (Vogt et al. 1991b), but subsequent work has detected PBPs in female antennae also (Callahan et al. 2000; Györgyi et al. 1988; Steinbrecht et al. 1992; Vogt 2002; Zhang et al. 2001). PBPs of noctuid moths have been found to show similar expression levels between the sexes (Vogt 2005).

To study the function of OBPs in *D. tabulaeformis*, first, we reported the identification of three OBPs, including one PBP and two GOBPs; then, we analyzed the molecular characteristics and evolutionary relationships of OBP sequences from *D. tabulaeformis* and other species; finally, we evaluated and compared the tissue expressions and the ligand binding properties of the PBP and GOBPs of *D. tabulaeformis*. In view of the finding that the binding of OBPs to ligand is pH dependent (Horst et al. 2001; Leal et al. 2005; Liu et al. 2012a; Sun et al. 2012; Wojtasek and Leal 1999), we also investigated the affects of pH on ligand binding for each protein.

## Methods and Materials

### Insect Rearing and Tissue Collection

Pupae of *D. tabulaeformis* were collected in HeBei, China in July 2011. The collection was approved by the Bureau of Forestry of Pingquan County, Chengde City, Hebei Province, China. Pupae were reared in our laboratory at 26 ± 2 °C and 50 ± 10 % relative humidity with a 16L:8D photoperiod. After emergence, adults were sexed. Then, the antennae, heads (without antenna), thoraxes, wings, abdomens, and legs of male and female *D. tabulaeformis* were collected separately and frozen immediately in liquid nitrogen. Each sample contained tissue from at least five insects, and more than five sample replicates were prepared.

### Preparation of cDNA and Genome DNA Samples

Total RNA was isolated using an RNeasy Mini Kit (Qiagen, Valencia, CA, USA), following the manufacturer’s instructions. Single-stranded cDNA templates were synthesized using 1 μg of total RNA with oligo(dT) as the anchor primer. M-MLV Reverse Transcriptase (Promega, USA) was used for cDNA synthesis, with reactions conducted at 42 °C for 1 h, and then stopped by heating at 70 °C for 15 min. Genomic DNA was prepared from thoracic muscles by using animal DNA isolation reagent (TaKaRa, Dalian, Liaoning, China) (Zhu et al. 1993).

### Identification of PBP and GOBP Encoding cDNA

The cDNAs that encode the OBPs of *D. tabulaeformis* were PCR amplified from antennal cDNA of males. A pair of degenerate primers (Table S1) was designed based on the known PBP and GOBP sequences of other lepidopterans. Amplification was carried out under the following conditions: 94 °C for 3 min; nine cycles of 94 °C for 30 s, 65 °C for 30 s, and 72 °C for 30 s, with a decrease in annealing temperature of 1 °C per cycle; 25 cycles of 94 °C for 30 s, 55 °C for 30 s, and 72 °C for 30 s; and a final incubation for 8 min at 72 °C. The reaction was performed in 25 μl with 1 μl single-stranded cDNA, 2.0 mM MgCl_2_, 0.2 mM dNTPs, 0.5 μM of each primer, and 1 U *Taq* polymerase (Promega). The PCR products of 400–600 bp were sequenced, and the products were identified as PBP1, GOBP1, and GOBP2.

The rapid amplification of cDNA ends (RACE) procedure was employed to amplify the 5′ and 3′ ends of the coding regions using the SMART RACE cDNA Amplification Kit (Clontech, Mountain View, CA, USA) following the kit instructions. Gene-specific primers (GSP in Table S1) for 5′- and 3′-RACE were derived from the sequences of the PCR products. Amplification conditions were 94 °C for 2 min; 30 cycles of 94 °C for 30 s, 65 °C for 30 s; and 72 °C for 3 min. The PCR products were cloned and sequenced.

### Isolation and Analysis of Genomic DNA Sequences

Regions of the three OBP genes were PCR amplified from genomic DNA using gene-specific primer pairs (Table S1) to ascertain its exon/intron structure. Touchdown PCR reactions were performed as follows: 94 °C for 3 min; nine cycles of 94 °C for 30 s, 65 °C for 30 s, and 72 °C for 2 min, with a decrease in annealing temperature by 1 °C per cycle; 34 cycles of 94 °C for 30 s, 55 °C for 30 s, and 72 °C for 2 min; and an incubation for 8 min at 72 °C. The PCR products were cloned and sequenced.

### Quantitative Real-time PCR

Specific primer pairs (Table S1) were derived from the cDNA sequences, and primer pairs for each gene were designed to amplify a 100–200 bp product, which was verified by sequencing. Normal RT-PCR using r*Taq* DNA polymerase (TaKaRa, Dalian, Liaoning, China) were conducted with each primer pair before quantitative Real-time PCR to ensure that the correct products were amplified and no primer dimers were present. Real-time PCRs were carried out in an Mx 3000P detection system (Stratagene, La Jolla, CA, USA) as described previously (Zhang et al. 2012), with thermal cycler parameters of 2 min at 95 °C; 40 cycles of 20 s at 95 °C, 20 s at 58 °C, and 20 s at 72 °C; and one cycle of 30 s at 95 °C, 30 s at 58 °C, and 30 s at 95 °C. β-Actin was used as the housekeeping gene. A standard curve was derived from ten-fold serial dilutions of plasmid containing the target DNA segment to determine the PCR efficiencies and for quantifying the amount of target mRNAs. All primers tested gave amplification efficiencies of 90–100 %. Five independent biological replicates (each biological replicate contained tissue from at least five insects) were performed for each tested item, and three technical replicates were performed for each reaction. The mRNA level of each gene then was quantified in relation to the expression of β-actin.

### Sequence Analyses

Sequences were identified by using the NCBI BLAST network server (Altschul et al. 1990). Putative signal peptides and their cleavage sites were predicted with the SignalP v.4.0 program (Dyrløv Bendtsen et al. 2004) (http://www.cbs.dtu.dk/services/SignalP/). The hydrophobicity profile was determined by the method of Kyte and Doolittle (1982). Sequences were aligned and compared using BioEdit (http://www.mbio.ncsu.edu/BioEdit/bioedit.html). The phylogenetic tree was constructed using the minimum evolution method with MEGA 5 (Tamura et al. 2011). Bootstrap analyses used 1,000 replications.

### Cloning of Expression Vectors

The crude PCR products encoding the mature protein, including the termination codon and flanked by the two restriction sites, *Nde*I and *Xho*1, were ligated into the pGEM-T vector (Promega, USA) after purification. Positive colonies were confirmed by DNA sequence. The resulting plasmids were digested by *Nde*I and *Xho*1 for 2 hr at 37 °C, and the digested product was separated by agarose gel electrophoresis. The obtained fragments was purified and ligated into the expression vector pET28b (Novagen, Darmstadt, Germany), which had been linearized with the same restriction enzymes. The resulting plasmid was sequenced to confirm that it encoded the mature protein.

### Expression and Purification of the Recombinant Protein

The recombinant vectors were transformed into BL21 DE3 *E. coli* cells. The positive clones were validated by PCR and sequencing. Protein expression was induced by addition of IPTG to a final concentration of 1 mM when the culture OD_600_ reached 0.6. Cells were grown for a further 4 hr at 30 °C, then harvested by centrifugation and sonicated. After centrifugation, the recombinant proteins were present as inclusion bodies. To solubilize them the proteins were dissolved in 8 M urea, 1 mM DTT in 50 mM Tris–HCl buffer, pH 7.4.

Purification of the proteins was performed with standard protocols previously adopted for other odorant-binding proteins (Ban et al. 2003b; Prestwich 1993), including affinity chromatography with a medium Ni Sepharose High Performance column (GE Healthcare, Little Chalfont, Buckinghamshire, UK). The protein renaturation and extensive dialysis were performed with a linear gradient of urea. This treatment does not affect the structure of insect OBPs, as previously demonstrated (Ban et al. 2003b; Guo et al. 2012). The protein production was with an N-terminal His tag, which was cleaved by thrombin (Sigma, USA). The cleaved proteins were purified again by the column mentioned above and stored at −70 °C until use.

### Fluorescence Measurements

Emission fluorescence spectra were performed on an F-4500 fluorescence Spectrophotometer (Hitachi, Japan) at 25 °C with a 1 cm light path quartz cuvette and 5 nm slits for both excitation and emission. The protein was dissolved in 50 mM Tris–HCl buffer, pH 7.4, while ligands used in the binding assay were added as 1 mM methanol solutions. The fluorescence of the mixture was recorded after 5 min to allow the signal to stabilize.

### Fluorescence Binding Assays

To measure the affinity of the fluorescent ligand 1-NPN to DtabPBP1, DtabGOBP1, and DtabGOBP2, a 2 μM solution of the protein in 50 mM Tris–HCl, pH 7.4, was titrated with aliquots of 1 mM 1-NPN dissolved in methanol to a final concentration of 16 μM. The probe was excited at 337 nm, and emission spectra were recorded between 380 and 450 nm. To evaluate the effect of pH on the binding affinity of the three OBPs, we also measured their binding with 1-NPN at a pH range of 4.5–9.0. The displacement of 1-NPN by selected ligands was measured in competitive binding assays using both the protein and 1-NPN at 2 μM. The mixtures were titrated with 1 mM methanol solutions of each competitor over concentration ranges of 2–16 μM. The fluorescence of the mixture was recorded after 5 min. Dissociation constants for 1-NPN and the stoichiometry of binding were obtained from Scatchard plots of the binding data through processing the data with Prism software. For other competitor ligands, the dissociation constants were calculated from the corresponding IC_50_ values using the equation: Ki = [IC50]/(1 + [1 − NPN]/K_1 − NPN_), where [1-NPN] is the free concentration of 1-NPN and K_1-NPN_ is the dissociation constant of the protein complex/1-NPN.

## Results

### Identification and Characterization of PBP1, GOBP1, and GOBP2 cDNA from D. tabulaeformis

The PCR using degenerate primers was designed based on the conserved amino acid regions in PBPs, GOBP1, and GOBP2 genes of other Lepidopterans. The reverse-transcription-PCR amplification yielded cDNA products that we named *DtabPBP1* (381 bp), *DtabGOBP1* (302 bp), and *DtabGOBP2* (266 bp), respectively. To obtain their full-length sequences, RACE was performed. Gene-specific primers for 5′- and 3′-RACE were derived from the sequences of the three cDNA products. The sequences of *DtabPBP1* (Fig. S1), *DtabGOBP1* (Fig. S2), and *DtabGOBP2* (Fig. S3) were deposited in GenBank with the accession numbers JX275385, JX275383, and JX275384, respectively.

The full-length sequence of *DtabPBP1* was 626 bp, and it contained a 489-bp open reading frame (ORF) that encoded a 163 amino acid protein with a calculated molecular weight of 18,404 Da and an isoelectric point of 5.16. Signal peptide prediction selected the initial 23 amino acids, suggesting that DtabPBP1 is a secretion protein. The mature DtabPBP1 comprises 140 amino acids, with a molecular weight of 15,894 Da and an isoelectric point of 5.14. The complete *DtabGOBP1* cDNA was 1,167 bp in length and contained a 498-bp ORF that encodes a 166 amino acid protein, with the first 19 amino acids predicted to be a signal peptide. The calculated molecular weight of DtabGOBP1 was 19,261 Da, and the isoelectric point was 5.47. The mature protein contained 147 amino acids, with a calculated mass of 17,101 Da and an isoelectric point of 5.22. The full-length sequence of *DtabGOBP2* was 612 bp and contained a 480-bp ORF that encodes a 160 amino acid protein with a calculated mass of 18,172 Da and an isoelectric point of 5.00. The initial 20 amino acids were predicted to be a signal peptide, resulting in a mature protein that contains 140 amino acids, with a calculated mass of 16,047 Da and an isoelectric point of 5.08.

A characteristic structural feature of PBPs and GOBPs is the alternation of hydrophobic and hydrophilic regions, resulting in a typical hydropathy plot (Fig. S4). The hydropathy profiles of DtabPBP1, DtabGOBP1, and DtabGOBP2 were very similar; all displayed the characteristic hydrophobic and hydrophilic regions, and all contained five of each type.

### Characterization of PBP1, GOBP1, and GOBP2 Genomic Sequences from D. tabulaeformis

To understand the exon/intron structure of the genes, the three OBP genes were cloned from genomic DNA and sequenced. *DtabPBP1*, *DtabGOBP1*, and *DtabGOBP2* all contained two introns and had similar exon/intron structures. The two introns in *DtabPBP1* were located between E45 and M46 (intron 1, 1,732 bp) and within the codon for D105 (intron 2, 236 bp) (Fig. S1). The introns in *DtabGOBP1* were located within the codons for E41 (intron 1, 992 bp) and G102 (intron 2, 368 bp) (Fig. S2). The introns in *DtabGOBP2* were located within the codons for E41 (intron 1, 732 bp) and G102 (intron 2, 821 bp) (Fig. S3). The ends of all the introns had a typical GT-AG structure.

### Evolutionary Analysis of PBP1, GOBP1, and GOBP2 from D. tabulaeformis and Other Lepidoptera

**Fig. 1:**
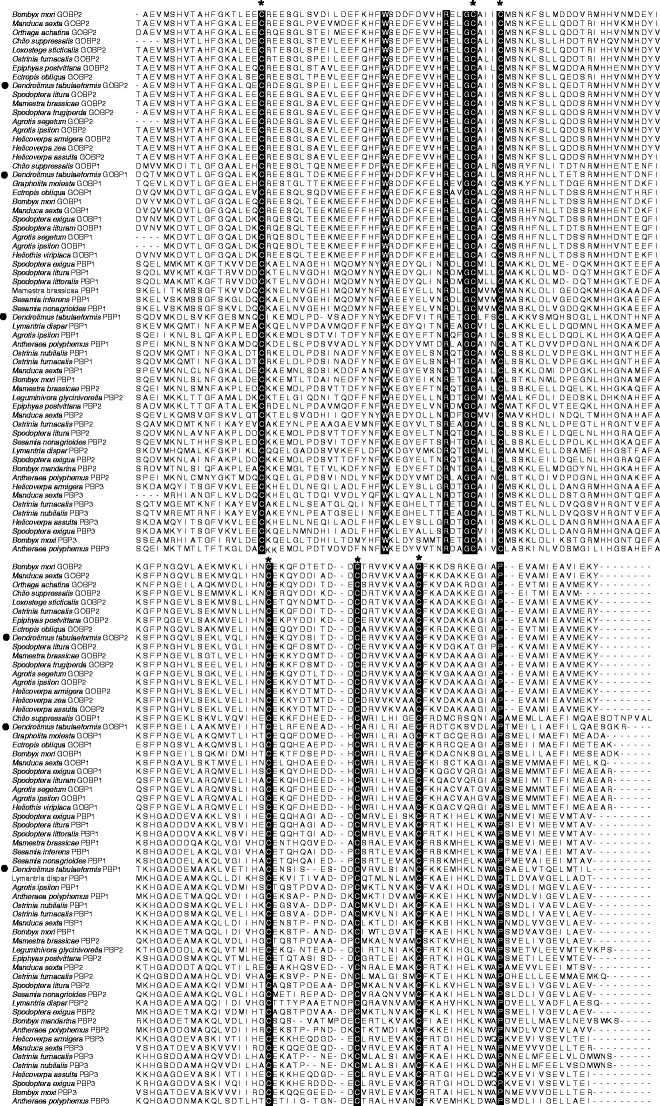
Alignments of the DtabPBP1, DtabGOBP1, and DtabGOBP2 mature amino acid sequences from *Dendrolimus tabulaeformis* with their orthologs from other insects. Conserved amino acids are shown with a black background. The positions of six conserved cysteine residues are indicated by asterisks

We aligned the mature amino acid sequences of DtabPBP1, DtabGOBP1, and DtabGOBP2 with their orthologs from other Lepidoptera species (Fig. 1). All three OBPs from *D. tabulaeformis* had conserved motifs characteristic of OBPs, including hydrophobic regions and six cysteine residues that form three disulfide bridges.

**Fig. 2:**
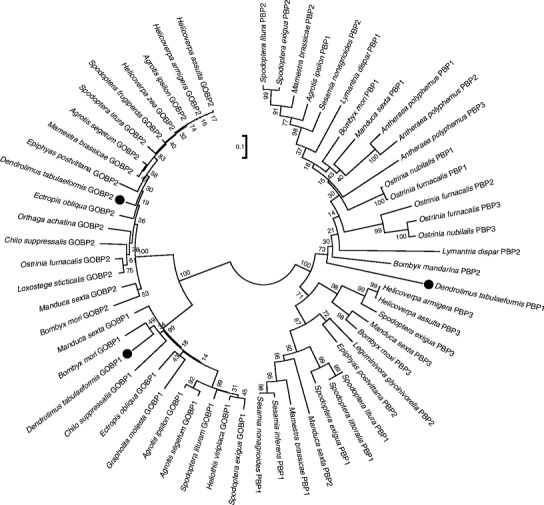
Minimum evolution tree of the DtabPBP1, DtabGOBP1, and DtabGOBP2 mature amino acid sequences and their orthologs from other insects. Bootstrap values from 1,000 replications indicate support for nodes. The bar indicates phylogenetic distance

We constructed a phylogenetic tree comparing mature DtabOBPs and OBPs from other lepidopteran insects (Fig. 2). In this tree, GOBP1, GOBP2, and PBP are well separated with strong bootstrap support. The distances among the PBPs seem much greater than that of GOBPs. Within the group of PBP1s, two clusters from various moths are apparent. *DtabPBP1* is located at the base of one cluster, neighboring *Bombyx mandarina*. The GOBP1 and GOBP2 sequences each formed an evolutionary grade, rather than two clades as PBP1s. The *D. tabulaeformis* GOBP1 was weakly supported as sister to *Bombyx mori* GOBP1, while the *D. tabulaeformis* GOBP2 was part of a polytomy within the GOBP2 sequences (Fig. 2).

### Tissue- and Sex-Dependent Distributions of OBPs from D. tabulaeformis

**Fig. 3:**
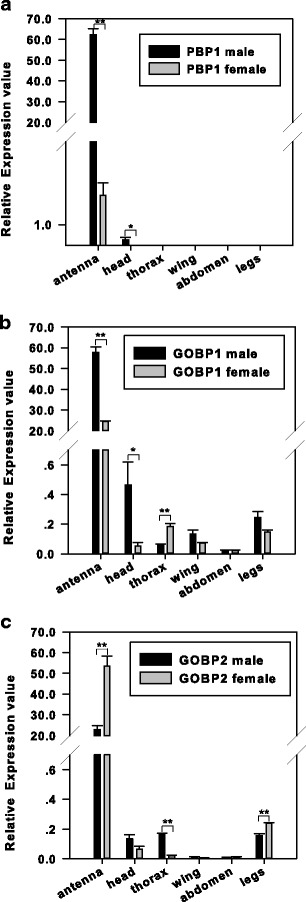
Tissue- and sex-dependent expression patterns of *DtabPBP1*, *DtabGOBP1*, and *DtabGOBP2*. Real-time PCRs were performed using RNA isolated from various tissues of male and female *Dendrolimus tabulaeformis*

Real-time PCR was performed to compare the tissue- and sex-dependent transcript levels of *DtabPBP1*, *DtabGOBP1,* and *DtabGOBP2* in *D. tabulaeformis. DtabPBP1* had its highest levels of expression in male antennae (about 62-fold higher than *β-Actin*), and the amount of *DtabPBP1* in female antennae was about 1.09 % of that in male antennae. *DtabPBP1* was not detectable in the thorax, abdomen, head (without antennae), wings, or legs of either sex, except that very low levels of *DtabPBP1* were detected in heads (without antennae) of male moths (Fig. 3a).

*DtabGOBP1* was also most highly expressed in the antennae of male *D. tabulaeformis* (about 58-fold higher than *β-Actin*). Its expression level in female antennae was 42.4 % of that in male antennae. Furthermore, *DtabGOBP1* was widely expressed in other body parts, such as the thorax, abdomen, head (without antennae), wings, and legs, especially in the heads of male moths. However, the expression levels in other body parts were much lower than those in the antennae (Fig. 3b).

In contrast to the other two genes, *DtabGOBP2* were most highly expressed in female antennae (about 53.6-fold higher than *β-Actin*). The expression levels of *DtabGOBP2* in male antennae were 42.68 % of those in female antennae. *DtabGOBP2* transcripts also were detected at low levels in the thorax, head (without antennae), and the legs of both sexes of *D. tabulaeformis* (Fig. 3c).

### Competitive Binding of Ligand to OBPs from D. tabulaeformis

**Fig. 4:**
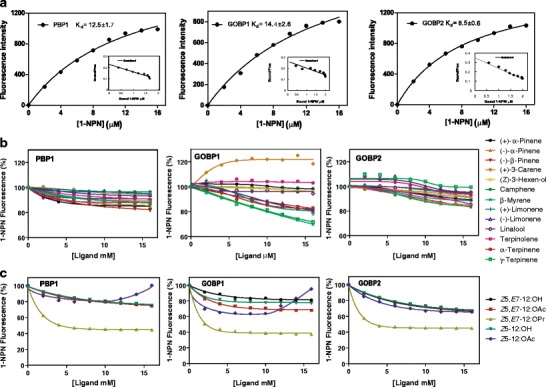
Competitive fluorescence binding assay of three DtabOBPs. **a** Binding curve of 1-NPN to three DtabOBPs and their resulting Scatchard plots. **b** Competitive binding curves of the selected host plant volatiles. **c** Competitive binding curves of the sex pheromone components of *Dendrolimus tabulaeformis*

To understand the ligand binding properties of the three OBPs, we prepared the recombinant proteins. Figure S5 reports the expression and purification of them. Then, we performed competitive ligand-binding experiments using well established protocols for other insect OBPs (Ban et al. 2002, 2003b; Guo et al. 2012; He et al. 2010; Zhou et al. 2009) to investigate the specificity of the binding pocket in the three *D. tabulaeformis* OBPs. The probe was excited at 337 nm, and emission spectra were recorded between 380 and 450 nm. The fluorescence probe 1-NPN alone produced weak fluorescence, then a strong blue shift, and a significant increase in fluorescence intensity was observed with the presence of OBPs. The three proteins DtabPBP1, DtabGOBP1, and DtabGOBP2 bind 1-NPN with dissociation constants 12.5 ± 1.7, 14.4 ± 2.6, and 8.5 ± 0.6, respectively (Fig. 4a). The pH value significantly affected the fluorescence of 1-NPN and different OBPs with concentration at 2 μM for both protein and 1-NPN. The binding curve indicates a rapid loss of affinity in acidic conditions, but similar affinity in basic buffers as in pH 7.4 (Fig. S6).

The displacement of 1-NPN by selected ligands was measured for the three OBP proteins. The ligands included the five potential sex pheromone components *Z*5,*E*7-12:OH, *Z*5,*E*7-12:OAc, *Z*5,*E*7-12:OPr, (*Z*)-5-dodecenyl alcohol (*Z*5-12:OH), and (*Z*)-5-dodecenyl acetate (*Z*5-12:OAc) (Kong et al. 2012), as well as the 13 main plant volatiles from the host of *D. tabulaeformis*, (+)-*α*-pinene, (−)-*α*-pinene, (−)-*β*-pinene, (+)-3-carene, (*Z*)-3-hexen-1-ol, camphene, *β*-myrene, (+)-limonene, (−)-limonene, linalool, terpinolene, *α*-terpinene, and *γ*-terpinene (Wang et al. 2005). These 13 plant volatiles were the most abundant of those released by the favorite host plant of *D. tabulaeformis*, *Pinus tabuliformis*, and the quantities of them change after tree damage (Liu 2007). The binding parameters for all compounds tested are listed in Table S2. The results of the fluorescence displacement of DtabPBP1 show that one of the pheromone component, *Z*5,*E*7-12:OPr, gave the most displacement (with K_i_ values of 3.61 μM), whereas the other sex pheromone components showed much less displacement (Fig. 4b). Consistent with other PBPs, the 13 plant volatiles showed only little displacement for DtabPBP1 (Fig. 4c).

GOBPs are postulated to be involved in perception of plant volatiles. β-Myrene and γ-terpinene displaced 30 % of the 1-NPN at a max concentration for DtabGOBP1, but DtabGOBP1 showed very low affinity for other volatiles; DtabGOBP2 also showed low affinity with the plant volatiles (Fig. 4b). Conversely, DtabGOBP1 and DtabGOBP2 displayed much higher binding affinities for the pheromone component *Z*5,*E*7-12:OPr, with K_i_ values of 1.9, and 2.7 μM, respectively (Fig. 4c).

## Discussion

Since the first insect OBP was found, many such proteins have been identified throughout Neoptera. Here, one PBP and two GOBPs were identified from the antennae of *D. tabulaeformis*. They all possess six conserved cysteine residues, which is characteristic for classical OBPs. Three interlocking disulfide bridges connect these six cysteine residues (Briand et al. 2001b; Leal et al. 1999). These data will enable future studies of the structure, function, and evolutionary relationships of these genes in related taxa.

The ligand-binding feature is the most important characteristic of OBPs, and has been the focus of many functional studies of OBPs (Ban et al. 2003a,b; Briand et al. 2001a; Campanacci et al. 2003; Maida et al. 2003; Pelosi et al. 2006; Vogt 2005; Zhou et al. 2009). PBPs have been demonstrated to bind pheromones in many species (Du and Prestwich 1995; Feixas et al. 1995; Maïbèche-Coisne et al. 1997). The sex pheromone compounds of *D. tabulaeformis* have been identified as *Z*5,*E*7-12:OH, *Z*5,*E*7-12:OAc, *Z*5,*E*7-12:OPr, *Z*5-12:OH, and *Z*5-12:OAc (Kong et al. 2012), but the two monoenes *Z*5-12:OAc and *Z*5-12:OH, alone or in binary mixtures, have no effect on the behavioral responses of *D. tabulaeformis*. Thus, *Z*5,*E*7-12:OAc, *Z*5,*E*7-12:OH, and *Z*5,*E*7-12:OPr are the key components of *D. tabulaeformis* sex pheromone, and the optimal ratio is 100:100:4.5. In this regard, DtabPBP1 may be tuned to detect one of these three key pheromone components, and we indeed observed a better affinity of DtabPBP1 to *Z*5,*E*7-12:OPr than to the other two sex pheromone components. Based on this observation, a possible explanation for the olfactory recognition mechanisms of *D. tabulaeformis* is that there are two other unidentified PBPs that may exhibit high binding affinities to *Z*5,*E*7-12:OAc and *Z*5,*E*7-12:OH, respectively, since most Lepidoptera contain more than one, and often three, PBP genes (Abraham et al. 2005; Maida et al. 2000). For example, in *Bombyx mori*, whose genome has been extensively analyzed, three PBPs were identified, but two (PBP2 and PBP3) are barely expressed (Zhou et al. 2009). Because Lasiocampidae is a sister family to Bombycidae (Kristensen et al. 2007), the other two PBPs proteins from *D. tabulaeformis* may also be weakly expressed like the PBP2 and PBP3 of *Bombyx mori*. They were difficult to be identified using the PCR strategy in this study. New gene identification strategies will be required in further investigations.

On the other hand, however, DtabGOBP1 and DtabGOBP2 also showed high affinity to *Z*5,*E*7-12:OPr but not to plant volatiles. This may indicate that although the proportion of *Z*5,*E*7-12:OPr is low in the sex pheromone of *D. tabulaeformis*, it plays a important role. This is consistent with the observation that traps baited with enough *Z*5,*E*7-12:OAc and *Z*5,*E*7-12:OH, but with little *Z*5,*E*7-12:OPr, were no better than unbaited controls (Kong et al. 2012). The pheromone binding feature of GOBPs, as shown in our results, has been found in several insects. For example, pheromone binding properties were found in CsupGOBP2 of *C. suppressalis* (Gong et al. 2009b), BmorGOBP2 of *B. mori* (He et al. 2010; Zhou et al. 2009), and AtraGOBP2 of *A. stransitella* (Liu et al. 2012b). Based on the fact that the adults of many species of moths, such as *D. tabulaeformis*, do not need to locate food or other resources, but focus mainly on mating and oviposition, we propose that the functions of OBPs in these moths may be due to convergent evolution. However, evidence is needed to support this proposition.

Some cases of abnormal fluorescence binding curves were observed in our results, such as the increase in fluorescence at high concentrations of *Z*5-12:OAc with DtabGOBP1, and DtabGOBP1 having increased 1-NPN fluorescence after the addition of (+)-3-carene. These findings may be due to the abnormal kinetics of these OBP-ligand mixtures. For some OBP-ligand interactions, 5 min may be insufficient time to establish an equilibrium (Gong et al. 2009a). The mathematical treatment that was used to extract K_i_ values assumed that: 1) the ligand and probe compete for one binding site and 2) the three partners (competing ligand, 1-NPN and PBP) reach equilibrium. These assumptions may not be met in the casess of DtabGOBP1 and (+)-3-carene, DtabPBP1 and *Z*5-12:OAc, and DtabGOPB1 and *Z*5-12:OAc.

In summary, we identified three new OBPs from an important forest pest, *D. tabulaeformis*. They are the first OBPs identified from Lasiocampidae. We analyzed their molecular characteristics, tested their tissue expression patterns, and identified their ligand binding affinities. The high affinity of *Z*5,*E*7-12:OPr to all three OBPs suggests that minor components of insect sex pheromones may play important roles.

## Electronic supplementary material

Below is the link to the electronic supplementary material.

Fig. S1 cDNA sequence and predicted amino acid sequence of DtabPBP1. The stop codon is indicated with an asterisk, the signal peptide is underlined, and the six conserved cysteines are boxed. The sites of introns one and two are marked with “><” under the sequence, and the intron sequences are given at the bottom of the figure. (PDF 2822 kb)

Fig. S2 cDNA sequence and predicted amino acid sequence of DtabGOBP1. The stop codon is indicated with an asterisk, the signal peptide is underlined, and the six conserved cysteines are boxed. The sites of introns one and two are marked with “><” under the sequence, and the intron sequences are given at the bottom of the figure. (PDF 2751 kb)

Fig. S3 cDNA sequence and predicted amino acid sequence of DtabGOBP2. The stop codon is indicated with an asterisk, the signal peptide is underlined, and the six conserved cysteines are boxed. The site of introns one and two are marked with “><” under the sequence, and the intron sequences are given at the bottom of the figure. (PDF 2514 kb)

Fig. S4 Predicted hydropathy profiles for the deduced amino acid sequences of DtabPBP1, DtabGOBP1, and DtabGOBP2. Hydropathy index values are plotted against the amino acid residues using the method of Kyte and Doolittle (1982) with a window size of nine amino acids. Positive values indicate hydrophobicity (PDF 177 kb)

Fig. S5 Bacterial expression and purification of DtabGOBP1, DtabGOBP2, and DtabPBP1. (A) 1, crude bacterial pellet of DtabGOBP1 before induction; 2-4, crude bacterial pellets of DtabGOBP1 after IPTG induction; M, markers; 5, crude bacterial pellet of DtabGOBP2 before induction; 6-8, crude bacterial pellets of DtabGOBP2 after IPTG induction. (B) 1, crude bacterial pellet of DtabPBP1 before induction; 2-4, crude bacterial pellets of DtabPBP1 after IPTG induction; M, markers. The proteins were obtained in high yield (about 20 mg/L of culture) as insoluble inclusion bodies and had to be denatured and renatured in order to be solubilized. (C) Fraction of DtabGOBP1 from chromatography purification. (D) Fraction of DtabGOBP2 from chromatography purification. (E) Fraction of DtabPBP1 from chromatography purification. (F) Proteins after His-tag cleavage by thrombin. 1, DtabGOBP1; 2, DtabGOBP2; 3, DtabPBP1; M, markers. Molecular weight markers (M) are 90, 66, 45, 35, 29, 20, 14.4 kDa. (GIF 92 kb)

High resolution image (TIFF 4045 kb)

Fig. S6 Effects of pH on the affinity of three *D. tabulaeformis* OBPs with 1-NPN. The concentration of different proteins and 1-NPN were both 2 μM. The relative fluorescence was calculated as a percentage of the fluorescence intensity at pH 9. (PDF 543 kb)

Table S1 (DOC 50 kb)

Table S2 (DOC 59 kb)
