# Supplementary material for: Molecular Characterization, Expression Pattern, and Ligand-Binding Property of Three Odorant Binding Protein Genes from Dendrolimus tabulaeformis
Source: J Chem Ecol. 2014 Apr 12;40(4):396–406. doi: 10.1007/s10886-014-0412-6 (PMC4008786; doi:10.1007/s10886-014-0412-6)
Supplement: Supplementary file 1 — cDNA sequence and predicted amino acid sequence of DtabPBP1. The stop codon is indicated with an asterisk, the signal peptide is underlined, and the six conserved cysteines are boxed. The sites of introns one and two are marked with “><” under the sequence, and the intron sequences are given at the bottom of the figure. (PDF 2822 kb) [file 10886_2014_412_MOESM1_ESM.pdf]

```
                                        TACATGGGGA CGCTACGAAA
 21 ATGACGAAGACTTATACGTTTTTAGCTGTAGCTATTGTCTTGTTG
    M  T  K  T  Y  T  F  L  A  V  A  I  V  L  L
 66 GCTATAGATTCAAGAGTTGATTCATCGCAGGATGTCATGAAAGAC
    A  I  D  S  R  V  D  S  S  Q  D  V  M  K  D
111 CTCAGCGTTAAGTTTGGAGAGTCAATGAATCAGTGCATTAAAGAG
    L  S  V  K  F  G  E  S  M  N  Q  C  I  K  E
                                                ><1
156 ATGGATCTTCCTGACGTGTCTGCGGACTTCTACAACTATTGGAAG
    M  D  L  P  D  V  S  A  D  F  Y  N  Y  W  K
201 GAGGATTTTGTTATTACGAGGCGTGAAACCGGCTGCCTTTTCTCA
    E  D  F  V  I  T  R  R  E  T  G  C  L  F  S
246 TGCCTTGCTAAGAAAGTGAGCATGCAGCA TCTGATGGACTGCTC
    C  L  A  K  K  V  S  M  Q  H  S  D  G  L  L
291 CACAAGGATAACACGCACAATTTCGCCACGAAACACGGCGCTGAT
    H  K  D  N  T  H  N  F  A  T  K  H  G  A  D
                                              ><2
336 GATGAAATGGCGGCCAAGCTGGTGGAAACCATCCACGCCTGCGAG
    D  E  M  A  A  K  L  V  E  T  I  H  A  C  E
381 AATTCAATTTCGGAGAGCGACGACTGCGTCCGGGTGCTGAGTATC
    N  S  I  S  E  S  D  D  C  V  R  V  L  S  I
426 GCGAACTGCTTCAAGAAGGAAATGCACAAACTGAACTGGGCACCC
    A  N  C  F  K  K  E  M  H  K  L  N  W  A  P
471 TCGGCGGAGCTTGTCACTCAAGAGCTGATGACAATCTTGTAA 512
    S  A  E  L  V  T  Q  E  L  M  T  I  L  *
                            TCACATCG CCCATCTAAG TCTCAAAAAC
AAATTCTTTT GCGCAATCAC ATATACAGTA GAACCTCTTT AAGTCAAAAA CGTCCCAAAA
AAAAAAAAAA AAAAAAAAAA AAAAAA
```

Intron 1

```
gtatgtcagt cattcagatt ccattagttt tttgttgaat gttttaatat atcaatactt
ctcatgtgtt gaaaatgtca atcatctaat aaaaatatgt gttgatggat aatatttgag
atcctaattc attggattaa agatcgacgt tgtgtaataa ttaaacgtaa cgtttgtatt
atttcattcc ttacaaaatt aagtagtaaa ccccaaaaaa tatgttcgag ttcctaagtc
ctatagtgtc acaactcatt taatttttaa atttctgtat tttattgttt gaaatatact
tttttagatc aatcaattaa cttaaaatat acctatataa taataaaaaa tatacctata
tacctcacca gataactgtt attgggcagt gtaacttgaa ggttagcagc attacctcgc
tagatacaaa ggtcaattct ttgagcgtag tccagtcttc cgttatcctc ttacgcgtgt
aaaaattgtg taatttcgtc gaagcaaaaa tgattttgct agtgaaatct tctttctaca
tttttaggat tttgaggtcc tgtctctaaa tttggaatta caagaaagaa aaattcaaga
cgaaagtttt ggcatcacag aattgaagag taactaattt aaatgatcat caatttaatc
atttttataa tttatgtctc aatatatttt tctaacgtct aaaatttaca ttattttttt
aacaaagcta tttaacccat ttatctcaca tgattcctct ttgtacatct gtcctttaac
gaagtataat ccatcttgga cactcttcaa ctcagaagca atggtagggt agtgttggac
ttccggtgac tttctcgaac ctactaacta tgtcactttt tgttacatca cgaatccgag
cctcagcgct ttgggaaaca aaaaccaaga atctattggt ttaatctatt ctctactcgt
aaatcgttga gtatctatac cctttccaag cattcatttt acttttgaca gcccgatgtg
catccagtta tgcattaccg actcgatttg ttataaaact cgatttcatt tacaaataag
ttttaatgaa ttatttttat atgacgtaga acatttcttc ttttaaaatc tttctttaga
tttatttact tatactgtcg atgttttgat agtatgcgcg ttacatgact cacaaaacat
ccttattgtg tcccacacaa caaaaaagta caacagtaat gtcatttgtt tcatgagggt
tttactatca agaaatttgt aactcgatca actgattgat agtatttttt tacatagcag
ctattttgga tcaaatacat atttttaatg aacagtagta accaaatgcc aaaattagtg
gacatttgtc acctcaccag aactcttaat tattactcga ttttatacaa ctgagaagtt
atacattaag aactattaac aggcttcaag tacatctaaa ttacttaaca tcgtgccatc
atgcattacc gctcatatac attgattcag gtactgcttc agggagcgga ctgactccaa
aatatttagc ctggaaagtc agccaggatt ggggaatcag tttttacacg attaccgtat
gaagtcaata ctagcttcct ttgaattttt agtcagtaga tagagtagac caatgtatat
cagagaattt caacgattgc ttctttgcta atacttctgc attttcattc ag
```

Intron 2

```
gtgagtactt tatactttca cagacaaagc acggcaccaa tattgctgaa atttgttagg
tatgtacgta gtattaatat caacgatgac attaagatat ttactatcga aaatgggata
atgtttccga gtagttttct ggagacaaaa agcgcctaga taaaaattaa atgcaatgtt
tagtgctttc aatataaact agggctaata ataaatatta ctgcatgttt gtttag
```
