# Supplementary material for: Molecular Characterization, Expression Pattern, and Ligand-Binding Property of Three Odorant Binding Protein Genes from Dendrolimus tabulaeformis
Source: J Chem Ecol. 2014 Apr 12;40(4):396–406. doi: 10.1007/s10886-014-0412-6 (PMC4008786; doi:10.1007/s10886-014-0412-6)
Supplement: Supplementary file 2 — cDNA sequence and predicted amino acid sequence of DtabGOBP1. The stop codon is indicated with an asterisk, the signal peptide is underlined, and the six conserved cysteines are boxed. The sites of introns one and two are marked with “><” under the sequence, and the intron sequences are given at the bottom of the figure. (PDF 2751 kb) [file 10886_2014_412_MOESM2_ESM.pdf]

```
                                      ACATGGGGAG TCGCCGCCGA GAGTCGACAA AA
 32 ATGCGCTGGACACAGCTGGCCCTGCTCGCGCTCTGCTGCCTGATG
    M  R  W  T  Q  L  A  L  L  A  L  C  C  L  M
 77 CAGGCCCGCGGGGACCAGACCGTCATGAAGGACGTCACCCTGGGA
    Q  A  R  G  D  Q  T  V  M  K  D  V  T  L  G
122 TTCGGGCAGGCCCTCGAAAAGTGCAGAGAGGAGAGTGGTCTGACA
    F  G  Q  A  L  E  K  C  R  E  E  S  G  L  T
                                         ><1
167 GACGAAAAGATGGAGGAGTTCTTTCATTTCTGGCATGACGACTTC
    D  E  K  M  E  E  F  F  H  F  W  H  D  D  F
212 AAATTTGTGCACCGTGAGCTTGGGTGCGCCATCCTCTGCATGAGC
    K  F  V  H  R  E  L  G  C  A  I  L  C  M  S
257 AGGCACTTCAACCTGCTCACGGAGACCAGCAGGATGCACCACGAG
    R  H  F  N  L  L  T  E  T  S  R  M  H  H  E
302 AACACAGACAACTTCATCAAGTCTTTCCCAACGGTGAGATCTTG
    N  T  D  N  F  I  K  S  F  P  N  G  E  I  L
                                           ><2
347 GCGGCGAAAATGGTGGAGATCATCCACACGTGCGAGCTGCGCTTC
    A  A  K  M  V  E  I  I  H  T  C  E  L  R  F
392 GAGAACGAGGCGGACCACTGCGCGCGCATCCTGCGCATCGCCGAG
    E  N  E  A  D  H  C  A  R  I  L  R  I  A  E
437 TGCTTCCGGGACACCTGCAAGTCCGTCGACCTCGCGCCCACCATG
    C  F  R  D  T  C  K  S  V  D  L  A  P  T  M
482 GAGATCCTCATAGCCGAATTCATCCTCCAGGCGGAGTCTGGCAAG
    E  I  L  I  A  E  F  I  L  Q  A  E  S  G  K
527 CGCTGA 532
    R  *
                                                            CCGCGACC
CAGGCCACCA CTTTCACCTG ACATCTATCC AATCACTTAT CTTTGTCGTT AACCAACCTT
CTGGTGCAAC TCCCGGCGGT GCTGACAGTC CGCAGTTTGT CTACTCCAGA ACTCATCTGT
TCCGACAGTC TCAGTACAAG TAATGTCAAT TTTCCTTCAA TATTAACATA AAATAATGAG
TAGTAAAATT AAGAAACTTG TCAAAAACAC GATATTATAG AACAAACTTA ACAAACAGAG
AACATTTGCC TTCAATCATA TATATCAAGT AGAAACAATT AATTATTATA TATTCAGTAT
AAATGAGGAT TGGTCTTATA TCTGAAAACA CACATCGCCG CAGTGTATTG TGTTACACTT
ATGTATATTT ATTTGGTATG AAAAATAGAC ACAAGTATTA TATTATTTGT CAACTTTTCA
ATTAATAGGG TACATTGTAA TATACTGTTT AAAAGATTTC AACGCGTATC CAGATTTTTA
ATTAGTTATT TTACTTGTAT TTTACATAAT TTTAAATGTG CATGTATTAT ATTAGCAATT
CATATCTAAT GCATATTATG TAAAAAATAT ATATATATGA AATATAAGAA ACAAAACTAA
AAAAAAAAAA AAAAAAAAAA AAAAAAA

Intron 1
gtcggttacc ctaccaatat ataagttgtc acgtatatca caacataggc accgacggct
ggtagttcat caacacctcc taaacacttg acaatcatct accagacaga cgtggcacct
gctcctttat gcctatttat gctggtcttt gtttacacaa cttttggaat ttttccagga
ccctgtaata tgaggttatt aaaaaaatac tataacaaat ttttgatgtt ccaaattagt
aggagcatca ccgccttcgt attgcagacc tccacttacc agcctgcctc tgctcatcag
caaacattaa aacttagtat tacgcgtatg ttgttgagaa ctcatactac taatccttac
aggtgttttg tttgtgttac gtactgtccg cgttagtctc ttttctacgc ctgaaggtaa
aacgttttaa atccttcctt tcttttgtct gttaattcat agaagccgta aagttcctaa
attgacaatt ccaaggctgg tgggcagagt tagacgggtt tcaactgcgt caccgtgtag
ttctgttgca ttcggttttg tttgtgacaa cagtgaatta cgtaacgttc ttctaataaa
ctttttaatc atttcaatgg cacatcatag attcacatcc cacaaattcg taacaatata
ttttgaatta aattccaatg tcaattaaat ctaggaagag gaaacaactc tcaaaataag
tagcttttct tgcttattga tgtgtgttat gtattaaata acaaacatca acgtagacct
tccaacattt actataatta tccacaatgg aatataaaag tgacataagt atatgtacat
atgaatctgc cccttttacga attataatca tgaatgtttt gagactctag ctcaatccaa
aacactttta tccaggagcg atgttaattt gagcggtctt gaagaaaggt tatagttatg
acacgtcttc taatacacta tttggtttac ag

Intron 2
gtaggacatt cttattcatt attcgctcac tcacgtcgaa ggtgggcggc tgatatcgat
ttagtagcta ctttggaatt agacaaaacc caatggatag tcttggagaa ggccttcgcg
ttatcgtcga gacaagctaa gaaggtttgc cgatatatat ttatcactca ctcattcaca
cttgtattgc gtcacctata aattgcctgt atattgttac aatgaagacg tgctgacgtt
gaaggaaaac acctccaagg gtcttgcaca ttgagataac tgacctgcta agcccgttga
ttgtgagagg tccgatccca gcaatgcgat attcatgtgc tgttcttgcg tttaggtgag
atctttag
```
