# Supplementary material for: Molecular Characterization, Expression Pattern, and Ligand-Binding Property of Three Odorant Binding Protein Genes from Dendrolimus tabulaeformis
Source: J Chem Ecol. 2014 Apr 12;40(4):396–406. doi: 10.1007/s10886-014-0412-6 (PMC4008786; doi:10.1007/s10886-014-0412-6)
Supplement: Supplementary file 3 — cDNA sequence and predicted amino acid sequence of DtabGOBP2. The stop codon is indicated with an asterisk, the signal peptide is underlined, and the six conserved cysteines are boxed. The site of introns one and two are marked with “><” under the sequence, and the intron sequences are given at the bottom of the figure. (PDF 2514 kb) [file 10886_2014_412_MOESM3_ESM.pdf]

```
                      ACATGGGGAT TATTGAAGAA CCAGTCAACA
 31 ATGTTGGTGTATCTGGTACCTTTGGTGATTGGGCTGGTGATGGAA
    M  L  V  Y  L  V  P  L  V  I  G  L  V  M  E
 76 CCGGTTGTTGGGACTGCAGAGGTGATGAGCCACGTGACAGCCCAC
    P  V  V  G  T  A  E  V  M  S  H  V  T  A  H
121 TTCGGGAAGGCTTTGCAGGAATGTCGTGATGAGTCAGGCCTGTCC
    F  G  K  A  L  Q  E  C  R  D  E  S  G  L  S
                                     ><
166 CCGGAGATCCTAGAGGAGTTCCAGCACTTCTGGAGCGAGGACTTC
    P  E  I  L  E  E  F  Q  H  F  W  S  E  D  F
211 GAGGTGGTGCACCGGGAGCTGGGCTGCGCCATCATCTGCATGTCG
    E  V  V  H  R  E  L  G  C  A  I  I  C  M  S
256 AACAAGTTCTCCCTCCTCCAGGAGGATACCCGGATGCATCATGTC
    N  K  F  S  L  L  Q  E  D  T  R  M  H  H  V
301 AACATGCACGACTATGTCAAGAGTTTCCCTAACGGCCAGGTCTTA
    N  M  H  D  Y  V  K  S  F  P  N  G  Q  V  L
                                      ><
346 TCAGAGAAGCTGGTGCAGCTGATTCACAATTGCGAGAAGCAGTAC
    S  E  K  L  V  Q  L  I  H  N  C  E  K  Q  Y
391 GACTCTATAACAGACGACTGTGAGCGTGTGGTGAAGGTCGCCGCG
    D  S  I  T  D  D  C  E  R  V  V  K  V  A  A
436 TGCTTCAAGGTGGACGCCAAGAAAGAGGGAATCGCCCCAGAAGTG
    C  F  K  V  D  A  K  K  E  G  I  A  P  E  V
481 GCCATGATCGAGGCCGTCATGGAGAAATACTGA 513
    A  M  I  E  A  V  M  E  K  Y  *
                                TCTACAA AATCTTCAAC AAAATCCACA
ATTCACTTAT ATTAAAATGT ATGGAATAAA TAAAGTTTAA CCTAAAAAAA AAAAAAAAAA
AAAAAAAAAA AA
```

Intron 1

```
gtaaggcatt ttcaaactcc tcaaataccg gaaaggttgg gagtaagata atcctcatca
tcttaccatg aaattgtggg tgcgttcgtt tcacgaaata taaagagttt cttagatgca
aatttcgtcg gtttactgca agaattcatt taactgatat acatagtata atatggcagt
taatgagctt ttgcagtaaa cggacgtttt ggtcaaaaaa ctacctaaat ggtacttgtt
tttccggtca gaatttgaac cggtcatctc taactaagct accatggttc aattatgtca
tcgtcatcat catccgagca tttaatcccg gttgcgttct gttatagata tattctatgc
atccatttca caccagtttt cattcagctt ccaatctaga cggtcatctc ttacaattga
tcattttatt ccaattattc tttttgaaa acgggttaac ctggttcctc gtagcaattc
ggagcgttca ctttaatgcc caaggtccgc tgacatcttc tctaatattt tatttttcgc
ggtttcatca tacgttacat atttctacta actatacagt agttcctgag aagcataaca
gttacttaca aggatccgat ggacgaacgg acccctacat gatgatttcg ttacaagtac
cattttaagc aagcctctaa gtattaattc aaatcttcgc agctattgat ataagtatat
tataaactcc ag
```

Intron 2

```
gtgggtttat ggatagggta aattatagta actagtggtc gttcgtggtc gaaattcgac
cataataatt tgctgtcgaa gactatactt ctataatcac agatttctcc aaggctacta
tacactttta atttgacttg aacaataaac aaaagagtat atatgcgtgt atgcgtcaaa
aacatggtag tgtgtgtaat atttttttat tgatttaata taatttgtat gcataattta
aaaaatatta gcagtcgtcc atttttctat atgcaaataa tgttaaatta cacttttttac
aactattttc aaaacactgt tcttataaaa gagcaagtgt taaaatgcag acaaaatgtc
atgagaatta aaaaaaaata tttaaacagt atgggcctta cttacatttt tttttaattt
aaagaatatg ctcggcaact gattagtgaa aatcctaccc ataaactcct ctttagtaga
atattttggg tacaacctac gttaatcatt gaaatttcgt ttatctgtgc tcctcttagg
taaacaacag gttggttaca tgggtataag tgaaataaaa agatattaat tgattagggt
ccgattctga ttctcttact taagtaatag tatcttaacg agattattat aagtaaggtc
atatgtgctt acgctatttc actccttaag tatgtgacgt ctccttgcgt tacgttaact
tgctacgccc ctattaaatc taggcggctt caacgtattg aaagatgata taaactacct
cagttgattc gactatctac cctgttcatg ttggattcca g
```
