# Supplementary figures and images for: Molecular Characterization, Expression Pattern, and Ligand-Binding Property of Three Odorant Binding Protein Genes from Dendrolimus tabulaeformis
Source: J Chem Ecol. 2014 Apr 12;40(4):396–406. doi: 10.1007/s10886-014-0412-6 (PMC4008786; doi:10.1007/s10886-014-0412-6)

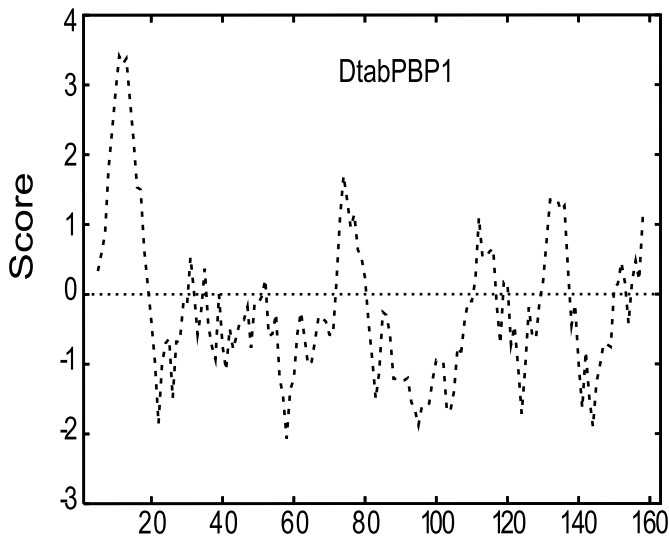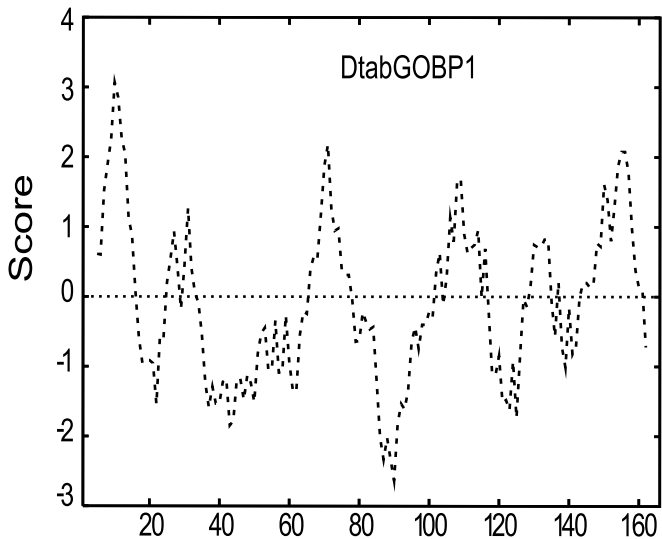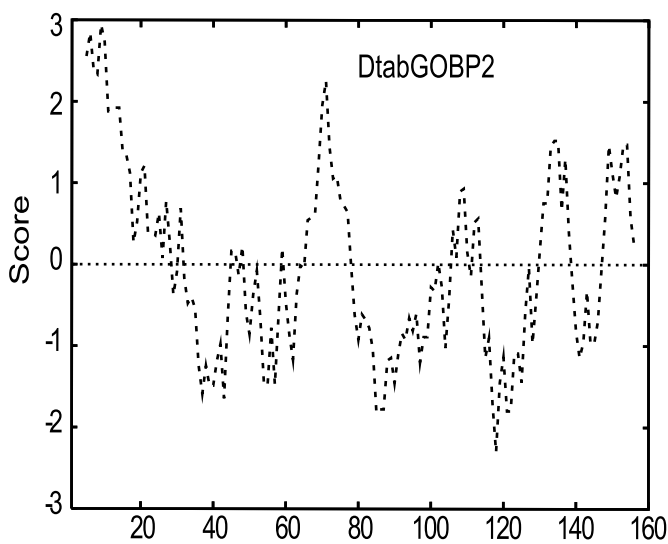

Supplement: Supplementary file 4 — Predicted hydropathy profiles for the deduced amino acid sequences of DtabPBP1, DtabGOBP1, and DtabGOBP2. Hydropathy index values are plotted against the amino acid residues using the method of Kyte and Doolittle (1982) with a window size of nine amino acids. Positive values indicate hydrophobicity (PDF 177 kb) [file 10886_2014_412_MOESM4_ESM.pdf]

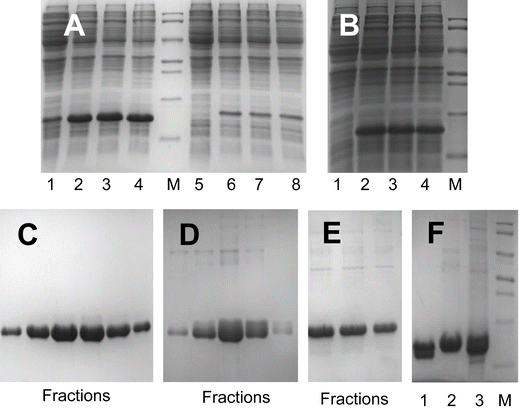

Supplement: Supplementary file 5 — Bacterial expression and purification of DtabGOBP1, DtabGOBP2, and DtabPBP1. (A) 1, crude bacterial pellet of DtabGOBP1 before induction; 2-4, crude bacterial pellets of DtabGOBP1 after IPTG induction; M, markers; 5, crude bacterial pellet of DtabGOBP2 before induction; 6-8, crude bacterial pellets of DtabGOBP2 after IPTG induction. (B) 1, crude bacterial pellet of DtabPBP1 before induction; 2-4, crude bacterial pellets of DtabPBP1 after IPTG induction; M, markers. The proteins were obtained in high yield (about 20 mg/L of culture) as insoluble inclusion bodies and had to be denatured and renatured in order to be solubilized. (C) Fraction of DtabGOBP1 from chromatography purification. (D) Fraction of DtabGOBP2 from chromatography purification. (E) Fraction of DtabPBP1 from chromatography purification. (F) Proteins after His-tag cleavage by thrombin. 1, DtabGOBP1; 2, DtabGOBP2; 3, DtabPBP1; M, markers. Molecular weight markers (M) are 90, 66, 45, 35, 29, 20, 14.4 kDa. (GIF 92 kb) [file 10886_2014_412_Fig5_ESM.gif]

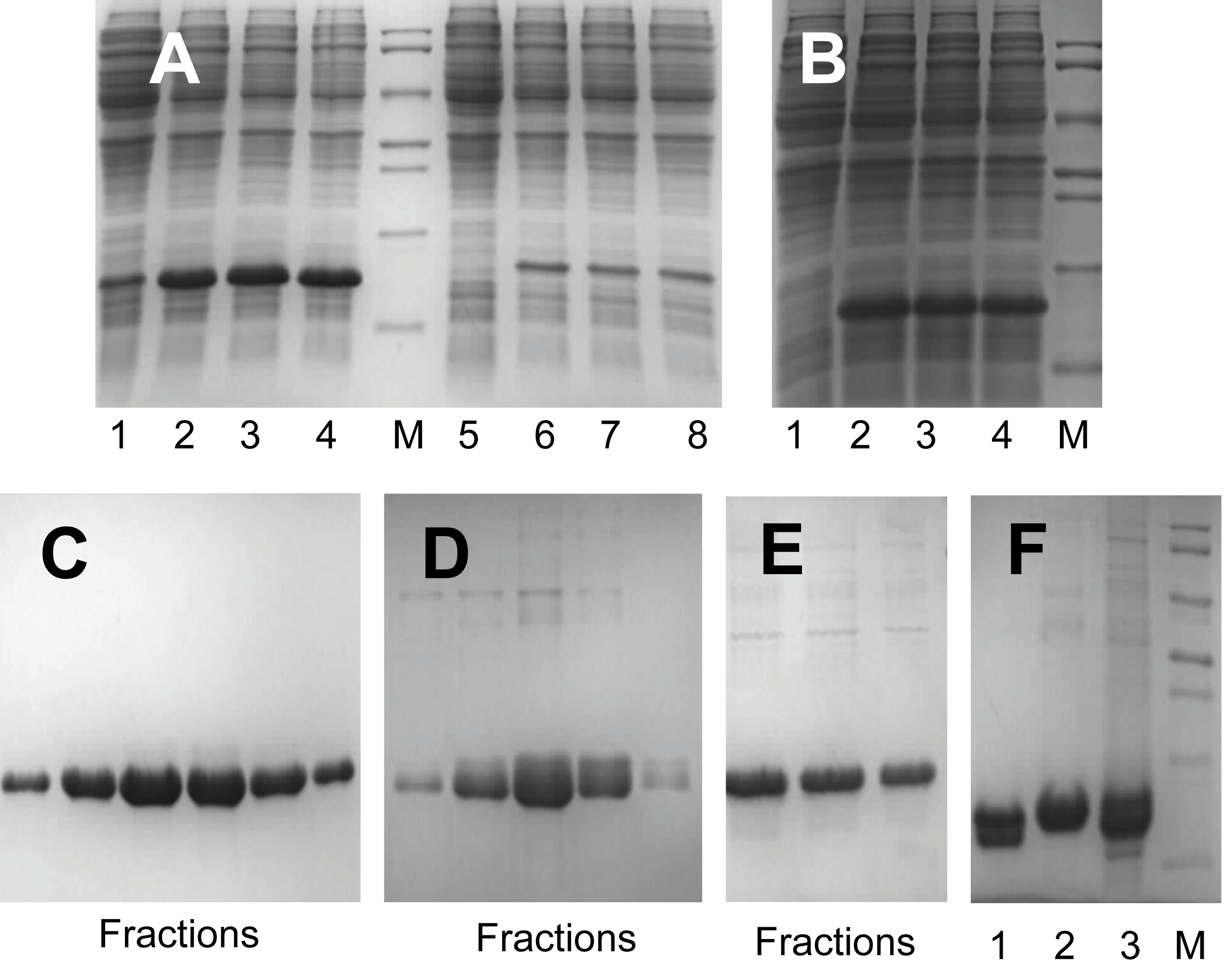

Supplement: Supplementary file 6 — High resolution image (TIFF 4045 kb) [file 10886_2014_412_MOESM5_ESM.tif]

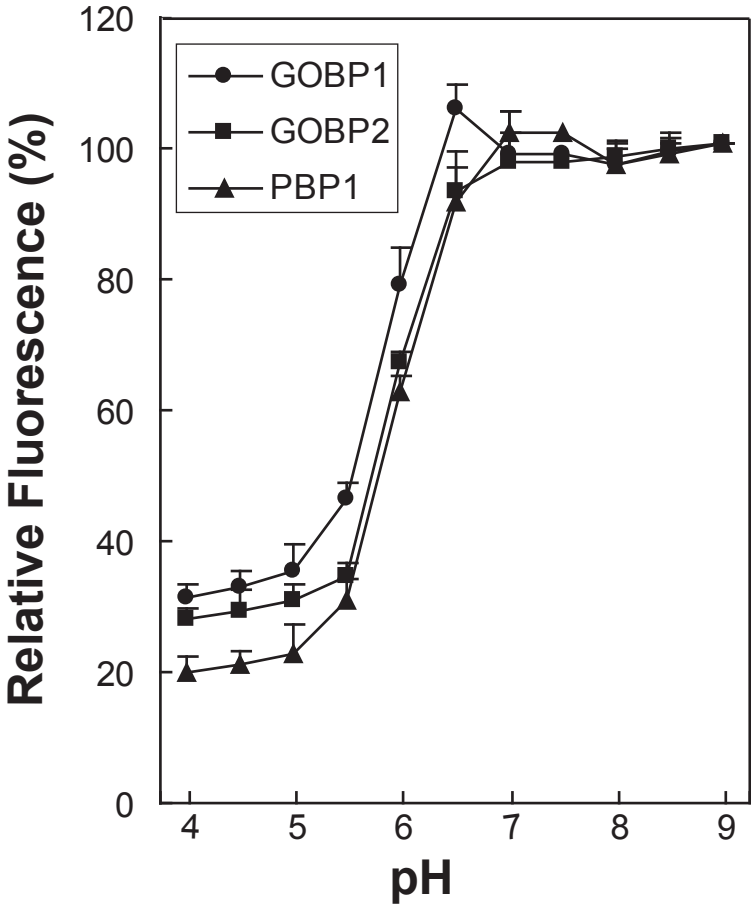

Supplement: Supplementary file 7 — Effects of pH on the affinity of three D. tabulaeformis OBPs with 1-NPN. The concentration of different proteins and 1-NPN were both 2 μM. The relative fluorescence was calculated as a percentage of the fluorescence intensity at pH 9. (PDF 543 kb) [file 10886_2014_412_MOESM6_ESM.pdf]
