# Supplementary material for: Molecular Characterization, Expression Pattern, and Ligand-Binding Property of Three Odorant Binding Protein Genes from Dendrolimus tabulaeformis
Source: J Chem Ecol. 2014 Apr 12;40(4):396–406. doi: 10.1007/s10886-014-0412-6 (PMC4008786; doi:10.1007/s10886-014-0412-6)
Supplement: Supplementary file 8 — (DOC 50 kb) [file 10886_2014_412_MOESM7_ESM.doc]

**Table S1.** OLIGONUCLEOTIDE PRIMERS USED FOR THE ISOLATION AND EXPRESSION ANALYSIS OF *Dendrolimus tabulaeformis* PBP1, GOBP1, And GOBP2

| **Purpose/Primer Name** | **Sequence (5'—3')** |
| --- | --- |
| **cDNA Isolation (RT-PCR)** | |
| *DtabPBP1*-5' | TCKCMAGARRTCATRAAGAAYTTA |
| *DtabPBP1*-3' | GTCCATGYYMGGMRCCCAGTTCA |
| *DtabGOBP1*-5' | GCTTYGGCCAGGCGCTCGACAAGTGC |
| *DtabGOBP1*-3' | TCGGMYTCCATGATGAACTC |
| *DtabGOBP2*-5' | GTGATGAGCCAYGTCACTGCDCATT |
| *DtabGOBP2*-3' | GCGTCYAYYTTGAAGCA |
| **5’ and 3’ cDNA End Isolation (RACE)** | |
| *DtabPBP1*-5'GSP | GGAAACCATCCACGCCTGCGAGAA |
| *DtabPBP1*-3'GSP | CACGCCTCGTAATAACAAAATCCTCCTTCC |
| *DtabGOBP1*-5'GSP | TCTTGGCGGCGAAAATGGTGGAG |
| *DtabGOBP1*-3'GSP | TCTCCACCATTTTCGCCGCCAAG |
| *DtabGOBP2*-5'GSP | CAGACGACTGTGAGCGTGTGGTGAAGG |
| *DtabGOBP2*-3'GSP | CGGCGACCTTCACCACACGCTCACAG |
| **Genomic DNA Isolation** | |
| *DtabPBP1*-5' | TGGGGACGCTACGAAAA |
| *DtabPBP1*-3' | AGGTTCTACTGTATATGTGATTGC |
| *DtabPBP1*-5'-2 | CGCTACGAAAATGACGAAG |
| *DtabPBP1*-3'-2 | AGCAAGGCATGAGAAAAGG |
| *DtabGOBP1*-5' | CCGAGAGTCGACAAAATGC |
| *DtabGOBP1*-3' | TTAAAAATCTGGATACGCG |
| *DtabGOBP2*-5' | GTGCGCCATCCTCTGCA |
| *DtabGOBP2*-3' | ATAAGTGAATTGTGGATTTTG |
| **Expression Analysis (Real-time PCR)** | |
| *DtabPBP1*-5' | TTCCTGACGTGTCTGCG |
| *DtabPBP1*-3' | ATTGTGCGTGTTATCCTTG |
| *DtabGOBP1*-5' | GTGCGCCATCCTCTGCA |
| *DtabGOBP1*-3' | GTGGTCCGCCTCGTTCT |
| *DtabGOBP2*-5' | GCACTTCTGGAGCGAGGAC |
| *DtabGOBP2*-3' | GTTAGGGAAACTCTTGACATAG |
| *DtabActin*-5' | TGCGTGACATCAAGGAGA |
| *DtabActin*-3' | AACGAGGGCTGGAAGAG |
