# Supplementary material for: Molecular Characterization, Expression Pattern, and Ligand-Binding Property of Three Odorant Binding Protein Genes from Dendrolimus tabulaeformis
Source: J Chem Ecol. 2014 Apr 12;40(4):396–406. doi: 10.1007/s10886-014-0412-6 (PMC4008786; doi:10.1007/s10886-014-0412-6)
Supplement: Supplementary file 9 — (DOC 59 kb) [file 10886_2014_412_MOESM8_ESM.doc]

**Table S2.** BINDING AFFINITIES OF LIGANDS TO RECOMBINANT *Dendrolimus tabulaeformis* ODORANT BINDING PROTEINS

| Ligands | DtabGOBP1 | | | DtabGOBP2 | | | DtabPBP1 | | |
| --- | --- | --- | --- | --- | --- | --- | --- | --- | --- |
|  | Max conc | %of max±SE | K_D_±SE (μM) | Max conc | %of max±SE | K_D_±SE (μM) | Max conc | %of max±SE | K_D_±SE (μM) |
| Pheromone components | | | | | | | | | |
| *Z*5,*E*7-12:OH | 16 | 81.0±1.0 |  | 16 | 65.6±0.8 |  | 16 | 74.5±0.6 |  |
| *Z*5,*E*7-12:OAc | 16 | 67.9±1.1 |  | 16 | 66.2±0.3 |  | 16 | 74.7±1.0 |  |
| *Z*5,*E*7-12:OPr | 16 | 37.1±0.5 | 1.9±0.2 | 16 | 44.8±0.7 | 2.7±0.3 | 16 | 44.5±0.6 | 3.6±0.3 |
| *Z*5-12:OH | 16 | 77.2±0.3 |  | 16 | 67.0±0.5 |  | 16 | 75.1±0.2 |  |
| *Z*5-12:OAc | 16 | Abnormal |  | 16 | 66.6±0.7 |  | 16 | Abnormal |  |
| Host plant volatiles | | | | | | | | | |
| (+)-α-Pinene | 16 | 98.3±2.2 |  | 16 | 88.8±2.2 |  | 16 | 83.9±0.3 |  |
| (-)-α-Pinene | 16 | 96.4±0.3 |  | 16 | 90.8±0.4 |  | 16 | 85.4±0.4 |  |
| (-)-β-Pinene | 16 | 82.8±1.0 |  | 16 | 83.2±1.1 |  | 16 | 80.7±0.5 |  |
| (+)-3-Carene | 16 | Abnormal |  | 16 | 84.3±0.9 |  | 16 | 85.3±1.7 |  |
| (*Z*)-3-Hexen-1-ol | 16 | 96.5±1.4 |  | 16 | 90.8±0.6 |  | 16 | 94.6±0.2 |  |
| Camphene | 16 | 96.7±1.2 |  | 16 | 93.8±0.7 |  | 16 | 96.5±0.2 |  |
| β-Myrene | 16 | 67.8±0.8 |  | 16 | 83.3±0.7 |  | 16 | 86.5±0.8 |  |
| (+)-Limonene | 16 | 78.2±1.1 |  | 16 | 88.0±1.3 |  | 16 | 94.4±0.4 |  |
| (-)-Limonene | 16 | 80.5±1.1 |  | 16 | 94.1±0.8 |  | 16 | 93.8±0.6 |  |
| Linalool | 16 | 90.4±0.9 |  | 16 | 95.2±0.6 |  | 16 | 90.4±0.7 |  |
| Terpinolene | 16 | 100.0±3.2 |  | 16 | 85.3±1.8 |  | 16 | 90.3±0.2 |  |
| α-Terpinene | 16 | 81.8±1.3 |  | 16 | 93.2±0.9 |  | 16 | 86.7±0.7 |  |
| γ-Terpinene | 16 | 71.8±0.7 |  | 16 | 99.3±0.4 |  | 16 | 88.1±0.3 |  |
